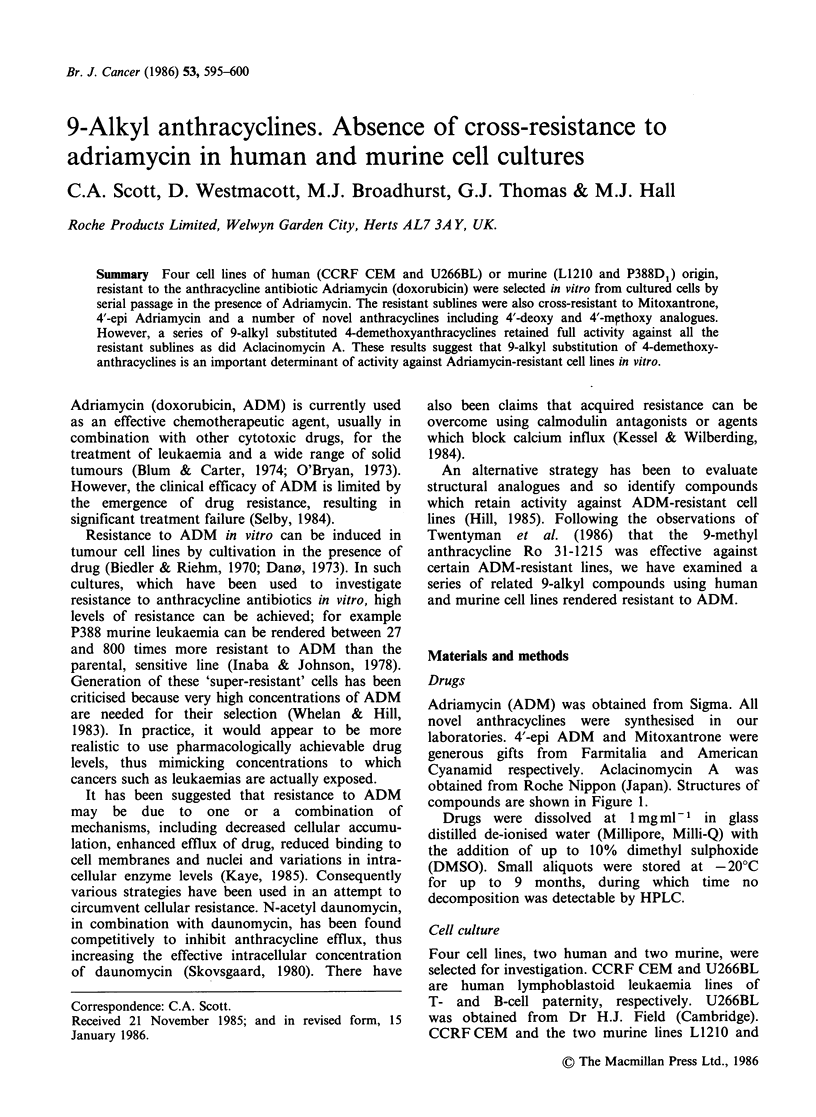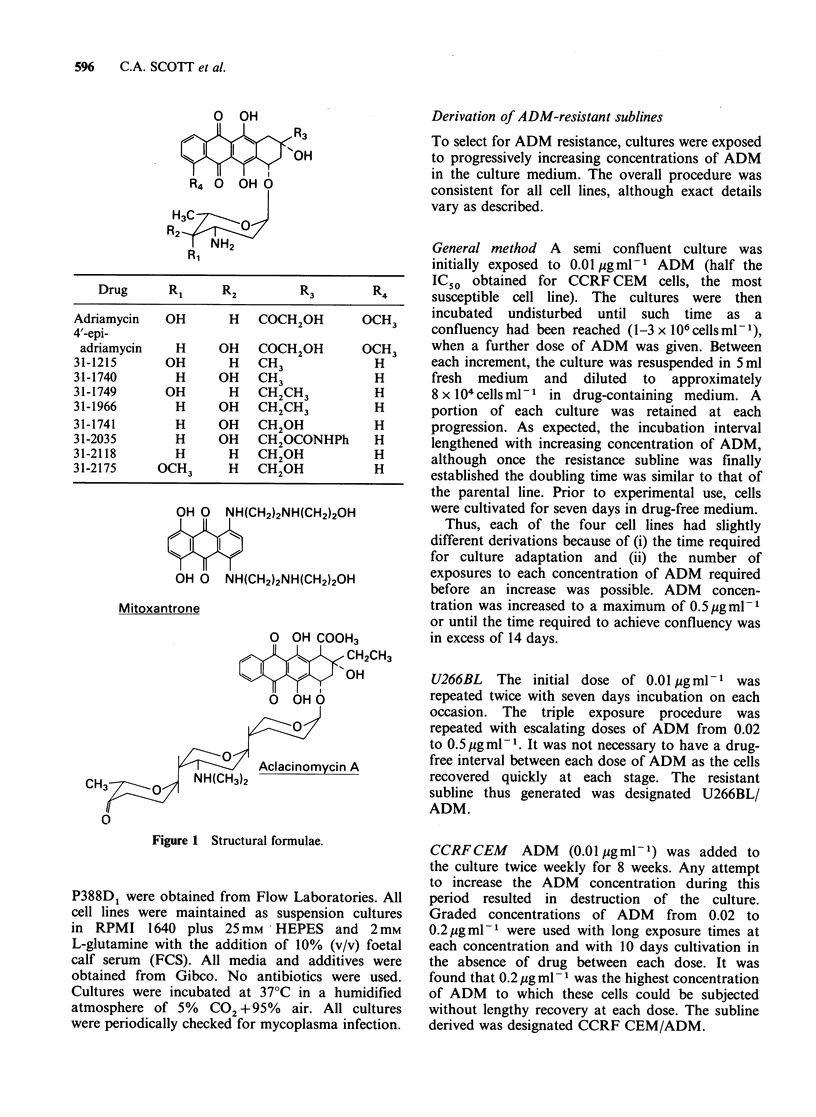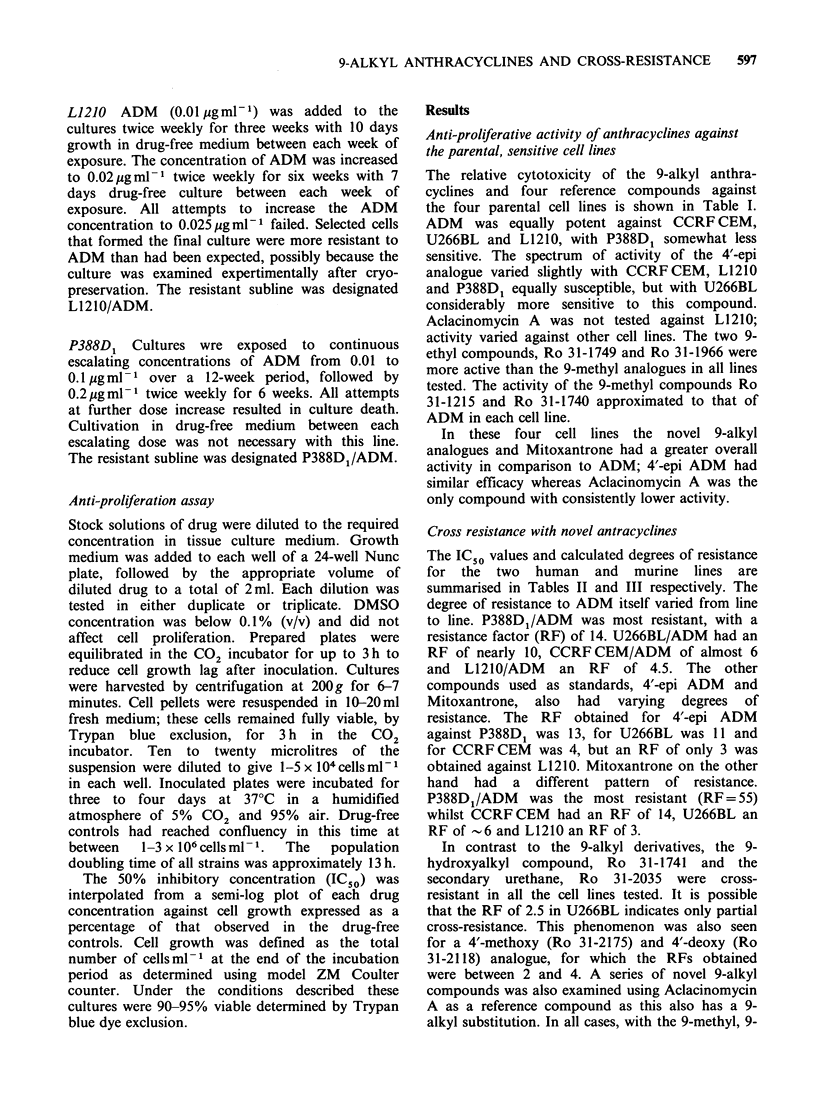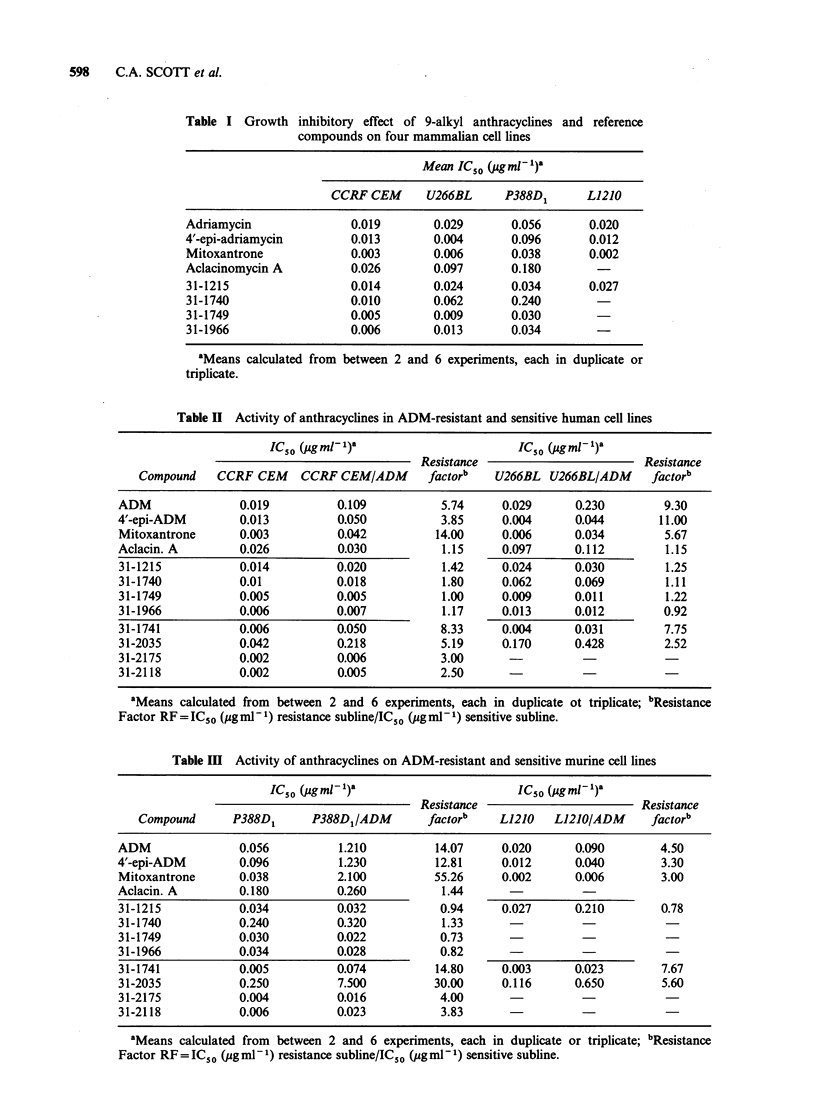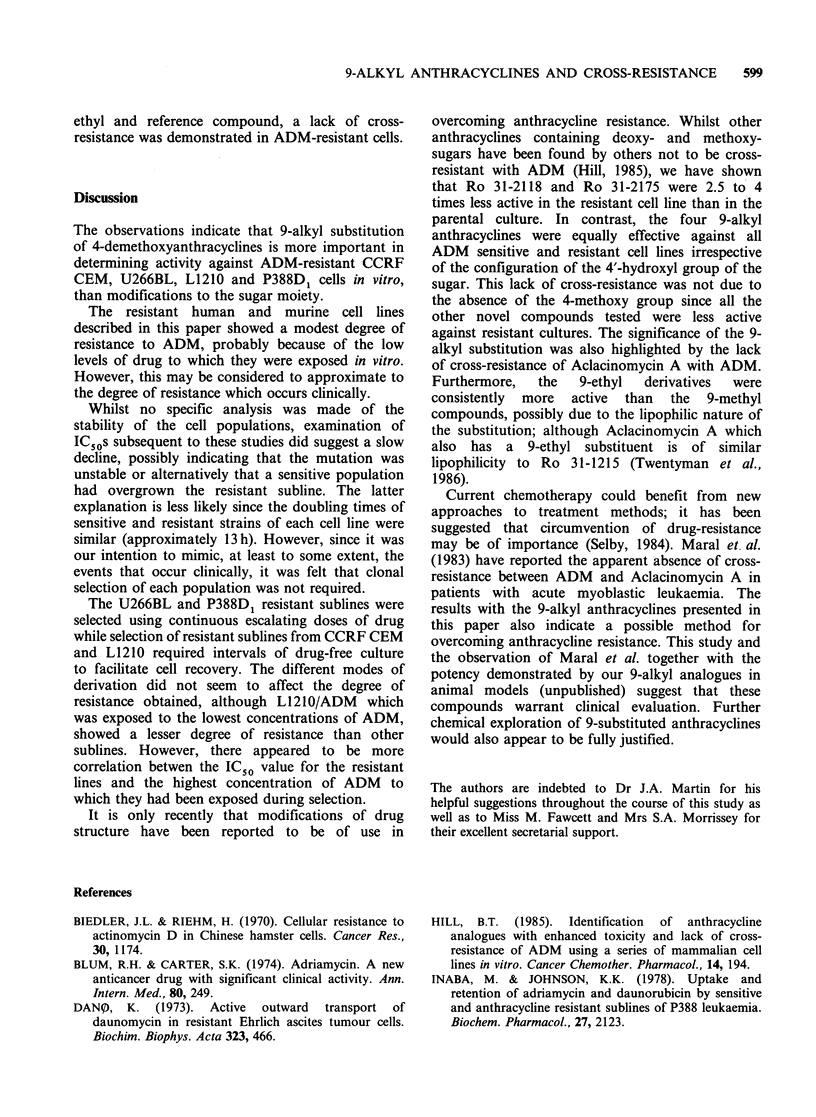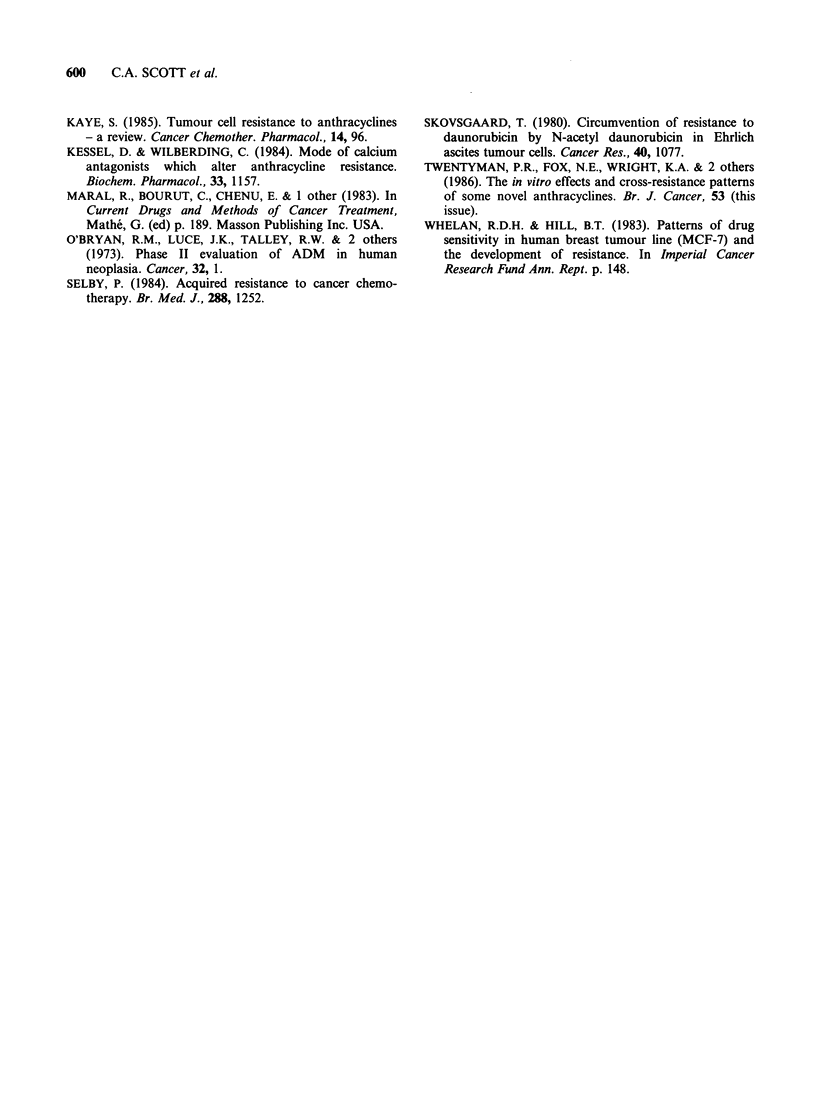# 9-alkyl anthracyclines. Absence of cross-resistance to adriamycin in human and murine cell cultures.

**DOI:** 10.1038/bjc.1986.101

**Published:** 1986-05

**Authors:** C. A. Scott, D. Westmacott, M. J. Broadhurst, G. J. Thomas, M. J. Hall

## Abstract

Four cell lines of human (CCRF CEM and U266BL) or murine (L1210 and P388D1) origin, resistant to the anthracycline antibiotic Adriamycin (doxorubicin) were selected in vitro from cultured cells by serial passage in the presence of Adriamycin. The resistant sublines were also cross-resistant to Mitoxantrone, 4'-epi Adriamycin and a number of novel anthracyclines including 4'-deoxy and 4'-methoxy analogues. However, a series of 9-alkyl substituted 4-demethoxyanthracyclines retained full activity against all the resistant sublines as did Aclacinomycin A. These results suggest that 9-alkyl substitution of 4-demethoxy-anthracyclines is an important determinant of activity against Adriamycin-resistant cell lines in vitro.


*Br. J. Cancer* (1986) **53**, 595–600

### 9-Alkyl anthracyclines. Absence of cross-resistance to adriamycin in human and murine cell cultures

C.A. Scott, D. Westmacott, M.J. Broadhurst, G.J. Thomas & M.J. Hall

*Roche Products Limited, Welwyn Garden City, Herts AL7 3AY, UK.*

**Summary** Four cell lines of human (CCRF CEM and U266BL) or murine (L1210 and P388$D_1$) origin, resistant to the anthracycline antibiotic Adriamycin (doxorubicin) were selected *in vitro* from cultured cells by serial passage in the presence of Adriamycin. The resistant sublines were also cross-resistant to Mitoxantrone, 4′-epi Adriamycin and a number of novel anthracyclines including 4′-deoxy and 4′-methoxy analogues. However, a series of 9-alkyl substituted 4-demethoxyanthracyclines retained full activity against all the resistant sublines as did Aclacinomycin A. These results suggest that 9-alkyl substitution of 4-demethoxy-anthracyclines is an important determinant of activity against Adriamycin-resistant cell lines *in vitro*.

Adriamycin (doxorubicin, ADM) is currently used as an effective chemotherapeutic agent, usually in combination with other cytotoxic drugs, for the treatment of leukaemia and a wide range of solid tumours (Blum & Carter, 1974; O'Bryan, 1973). However, the clinical efficacy of ADM is limited by the emergence of drug resistance, resulting in significant treatment failure (Selby, 1984).

Resistance to ADM *in vitro* can be induced in tumour cell lines by cultivation in the presence of drug (Biedler & Riehm, 1970; Danø, 1973). In such cultures, which have been used to investigate resistance to anthracycline antibiotics *in vitro,* high levels of resistance can be achieved; for example P388 murine leukaemia can be rendered between 27 and 800 times more resistant to ADM than the parental, sensitive line (Inaba & Johnson, 1978). Generation of these 'super-resistant' cells has been criticised because very high concentrations of ADM are needed for their selection (Whelan & Hill, 1983). In practice, it would appear to be more realistic to use pharmacologically achievable drug levels, thus mimicking concentrations to which cancers such as leukaemias are actually exposed.

It has been suggested that resistance to ADM may be due to one or a combination of mechanisms, including decreased cellular accumulation, enhanced efflux of drug, reduced binding to cell membranes and nuclei and variations in intracellular enzyme levels (Kaye, 1985). Consequently various strategies have been used in an attempt to circumvent cellular resistance. N-acetyl daunomycin, in combination with daunomycin, has been found competitively to inhibit anthracycline efflux, thus increasing the effective intracellular concentration of daunomycin (Skovsgaard, 1980). There have also been claims that acquired resistance can be overcome using calmodulin antagonists or agents which block calcium influx (Kessel & Wilberding, 1984).

An alternative strategy has been to evaluate structural analogues and so identify compounds which retain activity against ADM-resistant cell lines (Hill, 1985). Following the observations of Twentyman *et al.* (1986) that the 9-methyl anthracycline Ro 31-1215 was effective against certain ADM-resistant lines, we have examined a series of related 9-alkyl compounds using human and murine cell lines rendered resistant to ADM.

Correspondence: C.A. Scott.
Received 21 November 1985; and in revised form, 15 January 1986.

### Materials and methods

### *Drugs*

Adriamycin (ADM) was obtained from Sigma. All novel anthracyclines were synthesised in our laboratories. 4′-epi ADM and Mitoxantrone were generous gifts from Farmitalia and American Cyanamid respectively. Aclacinomycin A was obtained from Roche Nippon (Japan). Structures of compounds are shown in Figure 1.

Drugs were dissolved at $1\,mg\,ml^{-1}$ in glass distilled de-ionised water (Millipore, Milli-Q) with the addition of up to 10% dimethyl sulphoxide (DMSO). Small aliquots were stored at $-20°C$ for up to 9 months, during which time no decomposition was detectable by HPLC.

### *Cell culture*

Four cell lines, two human and two murine, were selected for investigation. CCRF CEM and U266BL are human lymphoblastoid leukaemia lines of T- and B-cell paternity, respectively. U266BL was obtained from Dr H.J. Field (Cambridge). CCRF CEM and the two murine lines L1210 and

© The Macmillan Press Ltd., 1986

| Drug | $R_1$ | $R_2$ | $R_3$ | $R_4$ |
|---|---|---|---|---|
| Adriamycin | OH | H | $COCH_2OH$ | $OCH_3$ |
| 4′-epi-<br>adriamycin | H | OH | $COCH_2OH$ | $OCH_3$ |
| 31-1215 | OH | H | $CH_3$ | H |
| 31-1740 | H | OH | $CH_3$ | H |
| 31-1749 | OH | H | $CH_2CH_3$ | H |
| 31-1966 | H | OH | $CH_2CH_3$ | H |
| 31-1741 | H | OH | $CH_2OH$ | H |
| 31-2035 | H | OH | $CH_2OCONHPh$ | H |
| 31-2118 | H | H | $CH_2OH$ | H |
| 31-2175 | $OCH_3$ | H | $CH_2OH$ | H |

Mitoxantrone

**Figure 1** Structural formulae.

$P388D_1$ were obtained from Flow Laboratories. All cell lines were maintained as suspension cultures in RPMI 1640 plus 25 mM HEPES and 2 mM L-glutamine with the addition of 10% (v/v) foetal calf serum (FCS). All media and additives were obtained from Gibco. No antibiotics were used. Cultures were incubated at 37°C in a humidified atmosphere of 5% $CO_2$+95% air. All cultures were periodically checked for mycoplasma infection.

*Derivation of ADM-resistant sublines*

To select for ADM resistance, cultures were exposed to progressively increasing concentrations of ADM in the culture medium. The overall procedure was consistent for all cell lines, although exact details vary as described.

*General method* A semi confluent culture was initially exposed to 0.01 $\mu g\,ml^{-1}$ ADM (half the $IC_{50}$ obtained for CCRF CEM cells, the most susceptible cell line). The cultures were then incubated undisturbed until such time as a confluency had been reached ($1–3 \times 10^6$ cells $ml^{-1}$), when a further dose of ADM was given. Between each increment, the culture was resuspended in 5 ml fresh medium and diluted to approximately $8 \times 10^4$ cells $ml^{-1}$ in drug-containing medium. A portion of each culture was retained at each progression. As expected, the incubation interval lengthened with increasing concentration of ADM, although once the resistance subline was finally established the doubling time was similar to that of the parental line. Prior to experimental use, cells were cultivated for seven days in drug-free medium.

Thus, each of the four cell lines had slightly different derivations because of (i) the time required for culture adaptation and (ii) the number of exposures to each concentration of ADM required before an increase was possible. ADM concentration was increased to a maximum of 0.5 $\mu g\,ml^{-1}$ or until the time required to achieve confluency was in excess of 14 days.

*U266BL* The initial dose of 0.01 $\mu g\,ml^{-1}$ was repeated twice with seven days incubation on each occasion. The triple exposure procedure was repeated with escalating doses of ADM from 0.02 to 0.5 $\mu g\,ml^{-1}$. It was not necessary to have a drug-free interval between each dose of ADM as the cells recovered quickly at each stage. The resistant subline thus generated was designated U266BL/ADM.

*CCRF CEM* ADM (0.01 $\mu g\,ml^{-1}$) was added to the culture twice weekly for 8 weeks. Any attempt to increase the ADM concentration during this period resulted in destruction of the culture. Graded concentrations of ADM from 0.02 to 0.2 $\mu g\,ml^{-1}$ were used with long exposure times at each concentration and with 10 days cultivation in the absence of drug between each dose. It was found that 0.2 $\mu g\,ml^{-1}$ was the highest concentration of ADM to which these cells could be subjected without lengthy recovery at each dose. The subline derived was designated CCRF CEM/ADM.

*L1210* ADM (0.01 $\mu g ml^{-1}$) was added to the cultures twice weekly for three weeks with 10 days growth in drug-free medium between each week of exposure. The concentration of ADM was increased to 0.02 $\mu g ml^{-1}$ twice weekly for six weeks with 7 days drug-free culture between each week of exposure. All attempts to increase the ADM concentration to 0.025 $\mu g ml^{-1}$ failed. Selected cells that formed the final culture were more resistant to ADM than had been expected, possibly because the culture was examined expertimentally after cryopreservation. The resistant subline was designated L1210/ADM.

*$P388D_1$* Cultures wre exposed to continuous escalating concentrations of ADM from 0.01 to 0.1 $\mu g ml^{-1}$ over a 12-week period, followed by 0.2 $\mu g ml^{-1}$ twice weekly for 6 weeks. All attempts at further dose increase resulted in culture death. Cultivation in drug-free medium between each escalating dose was not necessary with this line. The resistant subline was designated $P388D_1$/ADM.

*Anti-proliferation assay*

Stock solutions of drug were diluted to the required concentration in tissue culture medium. Growth medium was added to each well of a 24-well Nunc plate, followed by the appropriate volume of diluted drug to a total of 2 ml. Each dilution was tested in either duplicate or triplicate. DMSO concentration was below 0.1% (v/v) and did not affect cell proliferation. Prepared plates were equilibrated in the $CO_2$ incubator for up to 3 h to reduce cell growth lag after inoculation. Cultures were harvested by centrifugation at 200 *g* for 6–7 minutes. Cell pellets were resuspended in 10–20 ml fresh medium; these cells remained fully viable, by Trypan blue exclusion, for 3 h in the $CO_2$ incubator. Ten to twenty microlitres of the suspension were diluted to give $1–5 \times 10^4$ cells $ml^{-1}$ in each well. Inoculated plates were incubated for three to four days at 37°C in a humidified atmosphere of 5% $CO_2$ and 95% air. Drug-free controls had reached confluency in this time at between $1–3 \times 10^6$ cells $ml^{-1}$. The population doubling time of all strains was approximately 13 h.

The 50% inhibitory concentration ($IC_{50}$) was interpolated from a semi-log plot of each drug concentration against cell growth expressed as a percentage of that observed in the drug-free controls. Cell growth was defined as the total number of cells $ml^{-1}$ at the end of the incubation period as determined using model ZM Coulter counter. Under the conditions described these cultures were 90–95% viable determined by Trypan blue dye exclusion.

### Results

*Anti-proliferative activity of anthracyclines against the parental, sensitive cell lines*

The relative cytotoxicity of the 9-alkyl anthracyclines and four reference compounds against the four parental cell lines is shown in Table I. ADM was equally potent against CCRF CEM, U266BL and L1210, with $P388D_1$ somewhat less sensitive. The spectrum of activity of the 4′-epi analogue varied slightly with CCRF CEM, L1210 and $P388D_1$ equally susceptible, but with U266BL considerably more sensitive to this compound. Aclacinomycin A was not tested against L1210; activity varied against other cell lines. The two 9-ethyl compounds, Ro 31-1749 and Ro 31-1966 were more active than the 9-methyl analogues in all lines tested. The activity of the 9-methyl compounds Ro 31-1215 and Ro 31-1740 approximated to that of ADM in each cell line.

In these four cell lines the novel 9-alkyl analogues and Mitoxantrone had a greater overall activity in comparison to ADM; 4′-epi ADM had similar efficacy whereas Aclacinomycin A was the only compound with consistently lower activity.

*Cross resistance with novel antracyclines*

The $IC_{50}$ values and calculated degrees of resistance for the two human and murine lines are summarised in Tables II and III respectively. The degree of resistance to ADM itself varied from line to line. $P388D_1$/ADM was most resistant, with a resistance factor (RF) of 14. U266BL/ADM had an RF of nearly 10, CCRF CEM/ADM of almost 6 and L1210/ADM an RF of 4.5. The other compounds used as standards, 4′-epi ADM and Mitoxantrone, also had varying degrees of resistance. The RF obtained for 4′-epi ADM against $P388D_1$ was 13, for U266BL was 11 and for CCRF CEM was 4, but an RF of only 3 was obtained against L1210. Mitoxantrone on the other hand had a different pattern of resistance. $P388D_1$/ADM was the most resistant (RF = 55) whilst CCRF CEM had an RF of 14, U266BL an RF of ~6 and L1210 an RF of 3.

In contrast to the 9-alkyl derivatives, the 9-hydroxyalkyl compound, Ro 31-1741 and the secondary urethane, Ro 31-2035 were cross-resistant in all the cell lines tested. It is possible that the RF of 2.5 in U266BL indicates only partial cross-resistance. This phenomenon was also seen for a 4′-methoxy (Ro 31-2175) and 4′-deoxy (Ro 31-2118) analogue, for which the RFs obtained were between 2 and 4. A series of novel 9-alkyl compounds was also examined using Aclacinomycin A as a reference compound as this also has a 9-alkyl substitution. In all cases, with the 9-methyl, 9-

**Table I** Growth inhibitory effect of 9-alkyl anthracyclines and reference compounds on four mammalian cell lines

| | *Mean $IC_{50}$ ($\mu g\,ml^{-1}$)*[a] | | | |
|---|---|---|---|---|
| | *CCRF CEM* | *U266BL* | *$P388D_1$* | *L1210* |
| Adriamycin | 0.019 | 0.029 | 0.056 | 0.020 |
| 4′-epi-adriamycin | 0.013 | 0.004 | 0.096 | 0.012 |
| Mitoxantrone | 0.003 | 0.006 | 0.038 | 0.002 |
| Aclacinomycin A | 0.026 | 0.097 | 0.180 | — |
| 31-1215 | 0.014 | 0.024 | 0.034 | 0.027 |
| 31-1740 | 0.010 | 0.062 | 0.240 | — |
| 31-1749 | 0.005 | 0.009 | 0.030 | — |
| 31-1966 | 0.006 | 0.013 | 0.034 | — |

[a]Means calculated from between 2 and 6 experiments, each in duplicate or triplicate.

**Table II** Activity of anthracyclines in ADM-resistant and sensitive human cell lines

| *Compound* | *$IC_{50}$ ($\mu g\,ml^{-1}$)*[a] | | *Resistance factor*[b] | *$IC_{50}$ ($\mu g\,ml^{-1}$)*[a] | | *Resistance factor*[b] |
|---|---|---|---|---|---|---|
| | *CCRF CEM* | *CCRF CEM/ADM* | | *U266BL* | *U266BL/ADM* | |
| ADM | 0.019 | 0.109 | 5.74 | 0.029 | 0.230 | 9.30 |
| 4′-epi-ADM | 0.013 | 0.050 | 3.85 | 0.004 | 0.044 | 11.00 |
| Mitoxantrone | 0.003 | 0.042 | 14.00 | 0.006 | 0.034 | 5.67 |
| Aclacin. A | 0.026 | 0.030 | 1.15 | 0.097 | 0.112 | 1.15 |
| 31-1215 | 0.014 | 0.020 | 1.42 | 0.024 | 0.030 | 1.25 |
| 31-1740 | 0.01 | 0.018 | 1.80 | 0.062 | 0.069 | 1.11 |
| 31-1749 | 0.005 | 0.005 | 1.00 | 0.009 | 0.011 | 1.22 |
| 31-1966 | 0.006 | 0.007 | 1.17 | 0.013 | 0.012 | 0.92 |
| 31-1741 | 0.006 | 0.050 | 8.33 | 0.004 | 0.031 | 7.75 |
| 31-2035 | 0.042 | 0.218 | 5.19 | 0.170 | 0.428 | 2.52 |
| 31-2175 | 0.002 | 0.006 | 3.00 | — | — | — |
| 31-2118 | 0.002 | 0.005 | 2.50 | — | — | — |

[a]Means calculated from between 2 and 6 experiments, each in duplicate ot triplicate; [b]Resistance Factor RF = $IC_{50}$ ($\mu g\,ml^{-1}$) resistance subline/$IC_{50}$ ($\mu g\,ml^{-1}$) sensitive subline.

**Table III** Activity of anthracyclines on ADM-resistant and sensitive murine cell lines

| *Compound* | *$IC_{50}$ ($\mu g\,ml^{-1}$)*[a] | | *Resistance factor*[b] | *$IC_{50}$ ($\mu g\,ml^{-1}$)*[a] | | *Resistance factor*[b] |
|---|---|---|---|---|---|---|
| | *$P388D_1$* | *$P388D_1$/ADM* | | *L1210* | *L1210/ADM* | |
| ADM | 0.056 | 1.210 | 14.07 | 0.020 | 0.090 | 4.50 |
| 4′-epi-ADM | 0.096 | 1.230 | 12.81 | 0.012 | 0.040 | 3.30 |
| Mitoxantrone | 0.038 | 2.100 | 55.26 | 0.002 | 0.006 | 3.00 |
| Aclacin. A | 0.180 | 0.260 | 1.44 | — | — | |
| 31-1215 | 0.034 | 0.032 | 0.94 | 0.027 | 0.210 | 0.78 |
| 31-1740 | 0.240 | 0.320 | 1.33 | — | — | — |
| 31-1749 | 0.030 | 0.022 | 0.73 | — | — | — |
| 31-1966 | 0.034 | 0.028 | 0.82 | — | — | — |
| 31-1741 | 0.005 | 0.074 | 14.80 | 0.003 | 0.023 | 7.67 |
| 31-2035 | 0.250 | 7.500 | 30.00 | 0.116 | 0.650 | 5.60 |
| 31-2175 | 0.004 | 0.016 | 4.00 | — | — | — |
| 31-2118 | 0.006 | 0.023 | 3.83 | — | — | — |

[a]Means calculated from between 2 and 6 experiments, each in duplicate or triplicate; [b]Resistance Factor RF = $IC_{50}$ ($\mu g\,ml^{-1}$) resistance subline/$IC_{50}$ ($\mu g\,ml^{-1}$) sensitive subline.

ethyl and reference compound, a lack of cross-resistance was demonstrated in ADM-resistant cells.

### Discussion

The observations indicate that 9-alkyl substitution of 4-demethoxyanthracyclines is more important in determining activity against ADM-resistant CCRF CEM, U266BL, L1210 and P388D$_1$ cells *in vitro*, than modifications to the sugar moiety.

The resistant human and murine cell lines described in this paper showed a modest degree of resistance to ADM, probably because of the low levels of drug to which they were exposed *in vitro*. However, this may be considered to approximate to the degree of resistance which occurs clinically.

Whilst no specific analysis was made of the stability of the cell populations, examination of IC$_{50}$s subsequent to these studies did suggest a slow decline, possibly indicating that the mutation was unstable or alternatively that a sensitive population had overgrown the resistant subline. The latter explanation is less likely since the doubling times of sensitive and resistant strains of each cell line were similar (approximately 13 h). However, since it was our intention to mimic, at least to some extent, the events that occur clinically, it was felt that clonal selection of each population was not required.

The U266BL and P388D$_1$ resistant sublines were selected using continuous escalating doses of drug while selection of resistant sublines from CCRF CEM and L1210 required intervals of drug-free culture to facilitate cell recovery. The different modes of derivation did not seem to affect the degree of resistance obtained, although L1210/ADM which was exposed to the lowest concentrations of ADM, showed a lesser degree of resistance than other sublines. However, there appeared to be more correlation betwen the IC$_{50}$ value for the resistant lines and the highest concentration of ADM to which they had been exposed during selection.

It is only recently that modifications of drug structure have been reported to be of use in overcoming anthracycline resistance. Whilst other anthracyclines containing deoxy- and methoxy-sugars have been found by others not to be cross-resistant with ADM (Hill, 1985), we have shown that Ro 31-2118 and Ro 31-2175 were 2.5 to 4 times less active in the resistant cell line than in the parental culture. In contrast, the four 9-alkyl anthracyclines were equally effective against all ADM sensitive and resistant cell lines irrespective of the configuration of the 4′-hydroxyl group of the sugar. This lack of cross-resistance was not due to the absence of the 4-methoxy group since all the other novel compounds tested were less active against resistant cultures. The significance of the 9-alkyl substitution was also highlighted by the lack of cross-resistance of Aclacinomycin A with ADM. Furthermore, the 9-ethyl derivatives were consistently more active than the 9-methyl compounds, possibly due to the lipophilic nature of the substitution; although Aclacinomycin A which also has a 9-ethyl substituent is of similar lipophilicity to Ro 31-1215 (Twentyman *et al.*, 1986).

Current chemotherapy could benefit from new approaches to treatment methods; it has been suggested that circumvention of drug-resistance may be of importance (Selby, 1984). Maral *et. al.* (1983) have reported the apparent absence of cross-resistance between ADM and Aclacinomycin A in patients with acute myoblastic leukaemia. The results with the 9-alkyl anthracyclines presented in this paper also indicate a possible method for overcoming anthracycline resistance. This study and the observation of Maral *et al.* together with the potency demonstrated by our 9-alkyl analogues in animal models (unpublished) suggest that these compounds warrant clinical evaluation. Further chemical exploration of 9-substituted anthracyclines would also appear to be fully justified.

The authors are indebted to Dr J.A. Martin for his helpful suggestions throughout the course of this study as well as to Miss M. Fawcett and Mrs S.A. Morrissey for their excellent secretarial support.

### References

BIEDLER, J.L. & RIEHM, H. (1970). Cellular resistance to actinomycin D in Chinese hamster cells. *Cancer Res.*, **30,** 1174.

BLUM, R.H. & CARTER, S.K. (1974). Adriamycin. A new anticancer drug with significant clinical activity. *Ann. Intern. Med.*, **80,** 249.

DANφ, K. (1973). Active outward transport of daunomycin in resistant Ehrlich ascites tumour cells. *Biochim. Biophys. Acta* **323,** 466.

HILL, B.T. (1985). Identification of anthracycline analogues with enhanced toxicity and lack of cross-resistance of ADM using a series of mammalian cell lines *in vitro*. *Cancer Chemother. Pharmacol.*, **14,** 194.

INABA, M. & JOHNSON, K.K. (1978). Uptake and retention of adriamycin and daunorubicin by sensitive and anthracycline resistant sublines of P388 leukaemia. *Biochem. Pharmacol.*, **27,** 2123.

KAYE, S. (1985). Tumour cell resistance to anthracyclines – a review. *Cancer Chemother. Pharmacol.*, **14**, 96.

KESSEL, D. & WILBERDING, C. (1984). Mode of calcium antagonists which alter anthracycline resistance. *Biochem. Pharmacol.*, **33**, 1157.

MARAL, R., BOURUT, C., CHENU, E. & 1 other (1983). In *Current Drugs and Methods of Cancer Treatment*, Mathé, G. (ed) p. 189. Masson Publishing Inc. USA.

O'BRYAN, R.M., LUCE, J.K., TALLEY, R.W. & 2 others (1973). Phase II evaluation of ADM in human neoplasia. *Cancer*, **32**, 1.

SELBY, P. (1984). Acquired resistance to cancer chemotherapy. *Br. Med. J.*, **288**, 1252.

SKOVSGAARD, T. (1980). Circumvention of resistance to daunorubicin by N-acetyl daunorubicin in Ehrlich ascites tumour cells. *Cancer Res.*, **40**, 1077.

TWENTYMAN, P.R., FOX, N.E., WRIGHT, K.A. & 2 others (1986). The *in vitro* effects and cross-resistance patterns of some novel anthracyclines. *Br. J. Cancer*, **53** (this issue).

WHELAN, R.D.H. & HILL, B.T. (1983). Patterns of drug sensitivity in human breast tumour line (MCF-7) and the development of resistance. In *Imperial Cancer Research Fund Ann. Rept.* p. 148.